# A governance model for integrated primary/secondary care for the health-reforming first world – results of a systematic review

**DOI:** 10.1186/1472-6963-13-528

**Published:** 2013-12-20

**Authors:** Caroline Nicholson, Claire Jackson, John Marley

**Affiliations:** 1 Discipline of General Practice, University of Queensland, Brisbane, Australia; 2 Mater/UQ Centre for Primary Health Care Innovation, Mater Health Services, Level 2 JP Kelly Building, Raymond Tce, South Brisbane, Qld 4101, Australia

**Keywords:** Primary/secondary integration, Governance, Health system

## Abstract

**Background:**

Internationally, key health care reform elements rely on improved integration of care between the primary and secondary sectors. The objective of this systematic review is to synthesise the existing published literature on elements of current integrated primary/secondary health care. These elements and how they have supported integrated healthcare governance are presented.

**Methods:**

A systematic review of peer-reviewed literature from PubMed, MEDLINE, CINAHL, the Cochrane Library, Informit Health Collection, the Primary Health Care Research and Information Service, the Canadian Health Services Research Foundation, European Foundation for Primary Care, European Forum for Primary Care, and Europa Sinapse was undertaken for the years 2006–2012. Relevant websites were also searched for grey literature. Papers were assessed by two assessors according to agreed inclusion criteria which were published in English, between 2006–2012, studies describing an integrated primary/secondary care model, and had reported outcomes in care quality, efficiency and/or satisfaction.

**Results:**

Twenty-one studies met the inclusion criteria. All studies evaluated the process of integrated governance and service delivery structures, rather than the effectiveness of services. They included case reports and qualitative data analyses addressing policy change, business issues and issues of clinical integration. A thematic synthesis approach organising data according to themes identified ten elements needed for integrated primary/secondary health care governance across a regional setting including: joint planning; integrated information communication technology; change management; shared clinical priorities; incentives; population focus; measurement – using data as a quality improvement tool; continuing professional development supporting joint working; patient/community engagement; and, innovation.

**Conclusions:**

All examples of successful primary/secondary care integration reported in the literature have focused on a combination of some, if not all, of the ten elements described in this paper, and there appears to be agreement that multiple elements are required to ensure successful and sustained integration efforts. Whilst no one model fits all systems these elements provide a focus for setting up integration initiatives which need to be flexible for adapting to local conditions and settings.

## Background

Quote: *“It seemed like quite a few people had pieces of the jigsaw but no-one had the picture on the box” *[1]*.*

Health care reform aimed at improving quality and efficiency by empowering and supporting the primary care sector to better engage with the rest of the health care system has become the Millennium catch cry of governments across Europe, North America, Australia and New Zealand (NZ) [2]. Seamless integration of care between sectors, continuity of chronic care for patients and families, care close to where people live and work - the claims for community benefit in this new way of working appear compelling [3,4]. However the framework to allow the system transformation required to achieve this has proven elusive.

The integration of care, an organising principle for care delivery that aims to improve patient care and experience through improved coordination [5], between primary and secondary care sectors has shown minimal change since the United Kingdom (UK) commenced the rhetoric, with a ‘primary care led NHS’, in the mid-1990s [6,7]. Despite the early promise of reductions in Emergency Department and hospital attendance with new models of integrated care via independent practice associations (IPAs) in NZ, a shift in policy has only now seen a government focus on ‘better, sooner, more convenient health care in the community’ [8]. In Australia, the National Health and Hospitals Reform Commission Report first recommended significant governance change as an important element in increasing the effectiveness and efficiency of health care delivery [9]. In turn, regional service integration was one of the five key building blocks in Australia’s First National Primary Health Care Strategy [10]. Key health care reform rest on improved integration of care between the primary and hospital sectors, as meso-level primary care organisations (Medicare Locals [11]) and geographically based networks to deliver specialised hospital services (Local Hospital Networks [12]) formed from 2011, are required to work much more closely in care planning and delivery. Effective governance models to create, support and maintain the delivery of quality care involving multiple providers across social and health sectors are critical.

### Why integrated governance is important

Fragmentation of health services has created a complex, rapidly changing, and often impersonal health system that is increasingly difficult and frustrating to navigate [13]. To ensure health systems are sustainable, safe, fair, and agile enough to respond to changing health needs, recommendations for change in governance models have been suggested [9]. Governments have described how secondary care will be brought together with primary care organisations to coordinate and integrate primary health care services, jointly aiming to better coordinate services within sectors, but, the processes to integrate primary with secondary care have not been articulated [14].

It is important to gain consensus about integration targets which must be put into a strategic framework and agreed by partners to fulfil common integration goals [15]. In turn, integration agendas must be underpinned by effective governance mechanisms that are appropriate to the undertaking, the stakeholders involved, and the scale of delivery [16,17].

*‘Integrated Governance is a collation of systems, processes and behaviours by which healthcare organisations lead, direct and control their functions in order to achieve organisational objectives, safety and quality of service and in which they relate to patients and carers, the wider community and partner organisations’ *[18]. p104.

For the goals of health reform to be realised meso-level organisations must work together to achieve co-ordinated and integrated primary/secondary healthcare services however there is a lack of evidence to suggest how this will be achieved. The aim of this work is to provide evidence to these organisations to inform their working together. The objective of this review is to synthesise the existing published literature and to identify predominant reoccurring themes noted in citations, to form a framework for integrated primary/secondary health care governance, applicable to an international community, which allow optimal linkage between meso-level organisations [13,14]. This information can be used to strengthen the link between evidence, policy development and program implementation.

## Methods

### Search strategy

A search of electronic databases was conducted using data specific search terms and validated methods for retrieval from PubMed (NCBI), MEDLINE (Ovid), CINAHL (Ebsco), the Cochrane Library (Wiley), Informit Health Collection (Informit), and web communication platform resources including, the Primary Health Care Research and Information Service (PHC RIS), the Canadian Health Services Research Foundation, European Foundation for Primary Care, European Forum for Primary Care, and Europa Sinapse. The search was conducted for studies published between 2006 and 2012 (and 2013 in-press on-line articles). Articles not published in the English language were excluded. The review also included the relevant ‘grey’ literature including policy documents, reports, program evaluation and similar documentation through websites including Australian Primary Health Care Research Institute, Australian Department of Health, The Nuffield Trust [19] and The King’s Fund [20].

Search terms included words or phrases relating to; governance, integration, system, regional, collaboration, partnership, coordination, co-ordination and continuum. The search strategy for PubMed is shown in Table 1 and was repeated for other databases. The reference lists of reviewed studies and review articles were also considered for further relevant studies.

**Table 1:** Search terms

**Database**	**Platform**	**Search terms**
**PubMed**	**NCBI**	#1 governance[Text Word]
		Limits: Publication Date from 2006 to 2012
		#2 Search integrat*[Text Word] OR regiona*[Text Word] OR system*[Text Word] OR partnership*[Text Word] OR coordinat*[Text Word] OR co-ordinat* OR continuum[Text Word]
		Limits: Publication Date from 2006 to 2012
		#1 AND #2
		#3 (“Health Services”[Mesh]) OR “Decision Making, Organizational”[Mesh]) OR “Efficiency, Organizational”[Mesh]) OR “Models, Organizational”[Mesh]) OR “Comprehensive Health Care”[Mesh]) OR “Delivery of Health Care, Integrated”[Mesh]) OR “Patient-Centered Care”[Mesh]) OR “Health Care Reform”[Mesh]) OR “Managed Care Programs”[Mesh]) OR “Program Evaluation”[Mesh]) OR “Quality Assurance, Health Care”[Mesh]
		Limits: Publication Date from 2006 to 2012
		#1 AND #3

Studies were included if they made reference to integrated health care, and either governance or system reform. We included studies undertaken in any country (no specifications required) and any study type (e.g. systematic reviews, comparative studies, randomised controlled trials, descriptive studies, intervention studies, narrative reviews).

All searches were designed and conducted in collaboration with an experienced search librarian. All citations were imported into an electronic bibliographic database (Endnote Version X5).

### Study selection and screening

**Figure 1:**
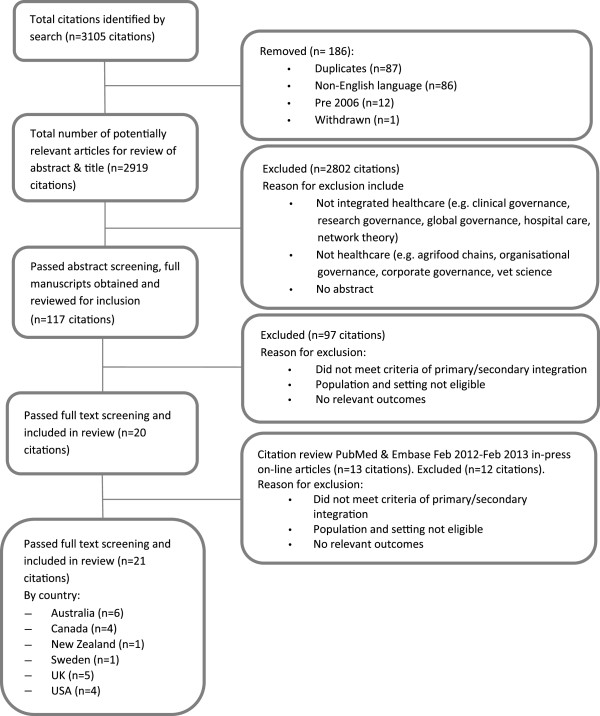
Process of systematic review.

One reviewer (CN) assessed all citations by title and abstract for potential relevance to the review. If there was any doubt to the relevance of the study or the abstract did not contain sufficient information for a decision it remained on the list. Results of screening were recorded against the citation in Excel spreadsheets. Full citations were ordered for all potentially relevant abstracts (n = 117). Full-text articles were reviewed by two reviewers (CN and SW), one with expertise in the area (CN), and were included if they fulfilled the inclusion criteria. To be included in the next review process papers had to be published in English, make reference to an aspect of integrated primary/secondary health care, and provide evidence of implementation, that is, the paper reported results of an evaluation study, case study or qualitative review of relevant current evidence. Outcomes were collected to demonstrate studies identified some impact from implementation. Outcomes reported related to care quality, efficiency and satisfaction, however, they were not synthesised for the purpose of this paper. A screening assessment was used to guide selection of relevant studies and results recorded on Excel spreadsheets for comparison purposes. If the reviewers were unable to reach a decision about whether to include or exclude, a third reviewer (CJ) was asked to review the article and make a decision. Articles meeting the eligibility criteria were included for data extraction (n = 21) (Figure 1).

We applied seven quality criteria for qualitative research (Table 2) [21]. Assessment of quality was not a criterion for exclusion however it gave insight into methods and limitations used for data collection and analysis in qualitative studies.

**Table 2:** **Criteria for assessing quality of qualitative research **[21]

**Does the research include:**	**Yes (score = 1)**	**No (score = 0)**
An explicit theoretical framework and/or literature review		
Aims and objectives are clearly stated		
A clear description of context		
A clear description of the sample and how it was recruited		
A clear description of methods used to collect and analyse data		
Attempts made to establish the reliability or validity of data analysis		
Inclusion of sufficient original or synthesised data to mediate between evidence and interpretation		

### Data extraction

A data extraction form was created to assist in systematically identifying main themes, methods, study design and setting. The main themes related to the key questions and included data collection on description of model characteristics (jurisdictions/sectors and/or organisations involved; duration/timeframe); measure(s) of effectiveness; reported outcomes; impact on patients/providers/policy makers/the system; and, reported barriers and enablers. Based on clinical and methodological expertise, one researcher (CN) was assigned to extract data from the eligible articles and the second (SW) reviewed the completed abstraction form alongside the original article for accuracy and completeness. Disagreements were settled by consensus or by obtaining a third reviewer’s opinion (CJ) if the first two investigators could not reach consensus. Data were entered into an excel spreadsheet whilst articles were being read.

One reviewer (CN) screened the bibliographies of all the key articles and reports and identified research articles and systematic reviews for inclusion. All additional articles and reviews identified through this snowballing process underwent the screening and data extraction process as detailed above.

### Data synthesis and analysis

Utilising research synthesis by configuration ‘entails the arrangement of thematically diverse individual findings … into a coherent theoretical rendering of them’ [22]. Top down synthesis allowed individual findings to be organised and previously unseen connections translated into a concept of theory [22]. Data was thematic analysed [23], organising data according to recurrent themes identified in the studies and key elements supporting integrated primary/secondary healthcare governance models were summarised. Text was free coded and a synthesis matrix was developed based on the themes (Table 3). This matrix was used to sort the data allowing it to be recorded, synthesised and compared.

**Table 3:** Themes identified in included studies

**First author**	**Year**	**Country**	**Population Focus (incl. enrolled populations)**	**Shared clinical priorities**	**Joint planning**	**Measurement**	**Innovation**	**Change Management**	**Professional development supporting value of joint working**	**Information Communication Technology**	**Incentives**	**Other**
Baker et al. [24]	2008	US	✓	✓		✓		✓	✓	✓	✓	
Connor et al. [25]	2010	UK	✓	✓	✓			✓	✓	✓		Patient engagement
Cumming [26]	2011	NZ	✓		✓						✓	
Featherstone [27]	2012	UK		✓						✓		
Fraschetti et al. [28]	2008	US			✓	✓		✓		✓	✓	
Ham [29]	2010	UK	✓	✓	✓	✓	✓	✓	✓	✓	✓	Patient engagement
Harris et al. [30]	2012	UK	✓	✓	✓			✓		✓	✓	
Hutchison et al. [31]	2011	Canada			✓							
Jackson et al. [32]	2008	Australia		✓	✓			✓	✓	✓	✓	
Jackson et al. [33]	2008	Australia		✓	✓	✓	✓	✓	✓	✓		
Jackson et al. [34]	2007	Australia	✓	✓	✓	✓	✓	✓	✓	✓		
Jackson et al. [16]	2008	Australia	✓	✓	✓			✓		✓	✓	
Jackson et al. [13]	2010	Australia	✓	✓	✓	✓	✓	✓	✓	✓	✓	Community engagement
Ovretveit et al. [35]	2010	Sweden	✓	✓	✓	✓		✓		✓	✓	Community engagement
Paulus et al. [36]	2008	US	✓	✓	✓	✓	✓	✓		✓	✓	Patient engagement
Peskett [18]	2009	UK		✓	✓	✓	✓	✓	✓			Patient and pubic engagement
Powell Davies et al. [37]	2008	Australia			✓				✓	✓	✓	
Rittenhouse et al. [38]	2009	US	✓			✓		✓			✓	
Smyth [17]	2009	Canada		✓	✓	✓		✓		✓	✓	
Suter et al. [39]	2009	Canada	✓	✓	✓	✓		✓	✓	✓	✓	Patient engagement
Wedel et al. [40]	2007	Canada	✓	✓	✓		✓	✓	✓	✓	✓	Community engagement

## Results and discussion

The search strategy identified a total of 3105 abstracts and titles, of which twenty-one studies met the inclusion criteria (Figure 1). The twenty-one papers included six from Australia, five from Canada, five from the UK, four from the United States (US), one from NZ, and one from Sweden. There were eleven case studies, one cross-sectional study, six reviews (including one perspective and one viewpoint) and three systematic reviews. All studies met 3 or more of the seven quality criteria for qualitative research, however only 5 of the 21 studies met all.

All studies were evaluations of the process of integrated governance and service delivery structures, rather than of service effectiveness. The evaluations included case reports (n = 17) and qualitative data analysis (n = 4). Ten studies (UK n = 4, Australia n = 2, NZ n = 1, Sweden n = 1, Canada n = 2) addressed policy change (e.g. *High Quality Care for All* and wave of Integrated Care Pilots in England), four from the US addressed business issues (e.g. cost containment through better care co-ordination and integration of operations), and seven (Australia n = 4, Canada n = 2, UK n = 1) addressed issues of clinical integration (e.g. care co-ordination between primary and secondary care). The relationship between these drivers was not examined.

### Elements of integrated primary/secondary systems

This systematic review identified ten elements necessary for integrated primary/secondary health care governance across a regional setting (Table 4). The specific interventions related to each element from the literature are outlined in detail below. Comparisons of how each element worked differently across settings was not included in this review.

**Table 4:** Elements of the integrated governance models identified in published papers (n = 21)

**Element**	**Interventions shown to be effective**	n = *[references]
1. Joint planning	Joint strategic needs assessment agreed; formalising relationships between stakeholders; joint boards; promotion of a community focus and organisational autonomy; guide for collective decision making; multi-level partnerships; focus on continuum of care with input from providers and users.	18
		[13,16-18,25,26,28-37,39,40]
2. Integrated information communication technology	Systems designed to support shared clinical exchange i.e. Shared Electronic Health Record; a tool for systems integration linking clinical processes, outcomes and financial measures.	17
		[13,16,17,24,25,27-30,32-37,39,40]
3. Change management	Managed locally; committed resources; strategies to manage change and align organisational cultural values; executive and clinical leadership; vision; commitment at meso and micro levels.	17
		[13,16-18,24,25,28-30,32-36,38-40]
4. Shared clinical priorities	Agreed target areas for redesign; role of multi-disciplinary clinical networks/clinical panels; pathways across the continuum.	16
		[13,16-18,24,25,27,29,30,32-36,39,40]
5. Incentives	Incentives are provided to strengthen care co-ordination e.g. pooling multiple funding streams and incentive structures, such as equitable funding distribution; incentives for innovative and development of alternative models.	15
		[13,16,17,24,26,28-30,32,35-40]
6. Population focus	Geographical population health focus.	13
		[13,16,24-26,29,30,34-36,38-40]
7. Measurement – using data as quality improvement tool	Shared population clinical data to use for planning, measurement of utilisation focusing on quality improvement and redesign; collaborative approach to measuring performance provides transparency across organisational boundaries.	12
		[13,17,18,24,28,29,33-36,38,39]
8. Continuing professional development supporting the value of joint working	Inter-professional and inter-organisational learning opportunities provide training to support new way and align cultures; clearly identifying roles and responsibilities and guidelines across the continuum.	11
		[13,18,24,25,29,32-34,37,39,40]
9. Patient/community engagement	Involve patient and community participation by use of patient narratives of experience and wider community engagement.	8
		[13,18,25,29,35,36,39,40]
10. Innovation	Resources are available and innovative models of care are supported.	7
		[13,18,29,33,34,36,40]

*Number of studies reporting the specified element.

### Joint planning

Eighteen studies indicated joint planning was a key element in developing integrated care across the primary/secondary care continuum. The studies indicated the following interventions are required to support joint planning:

– The ‘new way’ of working, including setting goals and strategies, as well as major decisions, are jointly determined and agreed by organisations across sectors [28,32,35,37]. A jointly agreed new approach to services, based on available hospital and primary care data, promoted flexible local health service delivery [13,25,28].

– Formal agreements between organisations and services have allowed them to move beyond the occasional informal partnership to a serious commitment to integrated health care and manage deliverables, risk and process through collaborative business approaches [16,30,37,39]. Examples include Integrated Care Trusts [25] and ‘Alliances’ [26] tasked with planning for geographical areas and to increase co-ordination between primary/secondary care.

– Joint board members with directors from primary and secondary care on each other’s boards [29,33,34] has facilitated greater appreciation of shared vision and values of organisations and the system as a whole. This has resulted in building trust and collaborative decision making [28].

– Shared planning needs a governance model that is community focused while preserving organisational autonomy of the individual health institutions/systems [16,17,28,29,40]. Decisions rendered must be in the best interest of the system. Shared planning processes based on shared principles have helped build trust, commitment and continuity through change [28,29].

– Collaborative decision-making has greatly facilitated understanding among all partners and, hence, supported change [17,40]. Although goodwill and focus on patient-centric care gets stakeholders to the table, once there they have to make decisions about how integration of services will be achieved [17]. A guide for collective decision making has assisted a collaborative approach to interagency working so interests and concerns are shared in an open and transparent way [17,30].

– Multi-level partnerships between clinician and management; between primary and secondary care that promote coordination across settings and levels of care are required [18,31,34,39].

– Understanding community need provides a starting point and a common aim. The challenge is to build capacity whilst respecting the role and reach of existing health service infra-structure [13,32,33,40].

– Providing opportunities for healthcare users and providers to come together and use information to arrive at a shared vision of optimal healthcare, has achieve clinician buy in and leadership while regaining professional autonomy [16,32,39,40].

– Planning for integrated services occurring across a region, settings and levels of care, including all core services along the continuum of health, for the population served is required [13,39]. Primary care organisations have provided a collective voice for this sector to address population need through planning and collaboration [26,31].

### Integrated information communication technology

Promotion of local integrated information communication technology (ICT) and e-connectivity was noted as a key element and significant enabler in integrating patient care across the continuum [13,24] and noted in seventeen studies. The most significant intervention was the shared electronic health record (SEHR) which supported integrated service development in the following ways:

– It is recognised as the beginning of a long care-transformation journey that requires a technical and physical infrastructure to deliver the expanded scope of practice [16,33,36].

– Has enabled the system as a whole to focus on the various ways in which they can better manage patient and population risk [25,30].

– Enabled acute, primary and community care providers to access more accurate and detailed clinical information to inform clinical decision making, for example, medication changes, BP changes over time, and reduced duplication [27,28,32,33,35].

– Is essential infrastructure for care co-ordination and communication across the continuum of care assisting in inter-professional communication across organisational boundaries – a move to ‘one patient, one chart’ [28,32,34,37,39,40].

– Supports consumer communication, and in some cases selected elements with limited data entry, via web portals supporting care closer to home [36,39]. In the US one consumer SEHR includes Internet-based lab results display and results trending over time, clinical reminders, self-scheduling, secure e-mail with providers, prescription refills, and educational content [36].

– Supports change management and provides evidence of impact and track changes in patient care [29,35].

– Has provided a useful way to manage performance and achieve high-quality healthcare improvement as it allows data management and effective tracking of utilization and outcomes [17,30,39,40].

In addition to the SEHR, studies suggest integrated ICT systems have been a clinical accelerator to improvement across the system by linking clinical processes, outcomes and financial measures [24]. This ability to integrate clinical and financial information, across health and social care, is viewed as important for monitoring cost-effectiveness, as well as for facilitating service planning, identifying high risk patients, and coordinating decision making across providers [29,30,39,40]. In the US one system reported not showing a return on investment for their SEHR until it was combined with their clinical improvement, here ‘informatics builds the tools, clinical quality improvement builds the content’ [24].

### Change management

Change management was also mentioned as a significant element in seventeen studies. Various authors recognised that having an effective change management strategy is the foundation that underpins integration work [32,35,40]. Change takes time, should be managed locally and requires committed resources for the development of processes and strategies that support implementation to be sustainable [17,28,29,32,34-36,39,40]. With strong and committed executive and clinical leadership it is possible to sustain partnerships and to deliver innovation and improvements in care [13,17,24,28-30,34,38-40]. Key individuals instrumental in providing support to integration initiatives have to step outside traditionally established boundaries. These people were committed to making a change and support others in creating needed change [17,40].

Linking change to an improvement agenda supports a culture across organisations which focus on high quality care [18,36]. Integrated governance is guided by a strong, jointly agreed vision to align efforts and conflicting aspirations of different parts of the system, shared and clear purpose and goals, and a revisiting of the mandate frequently keep a focus on the system [16-18,24,28,29,33,36]. Finally, early change management at meso-level is provided by organizational support with demonstration of commitment to integrate which in turn enables clinicians and managers to develop the ability to make change happen at the micro level [13,16,18,24,25,29,30,32,35,36,39,40].

### Shared clinical priority areas

Sixteen studies focused on shared clinical priority areas between organisations provided the targets for agreed redesign. In some cases health system leaders or multi-disciplinary clinical panels across primary/secondary care identified agreed clinical priority areas most likely to produce real impact, were quick to use data on service delivery and to prioritise key value added processes [13,16,18,24,25,27,29,30,32-34,36,39] whilst in other cases priorities were agreed from community assessment [40]. Multi-disciplinary groups/clinical networks involving all providers (primary, community, social and secondary care) supported a team based approach to developing integrated service delivery models, improved partnership working and improved care planning and coordination [16,30,33,34]; and, development of co-ordinated patient pathways for entire episodes of care, both optimise care across the continuum [17,32,35,36].

### Incentives aligned

The need to align incentives to support the clinical integration strategy was noted in fifteen studies [13,24,32,35,36,39]. One example in the US provided each clinical program with a set of core indicators that measure their own results against peer, regional and system-level results and goals, and practice groups could be financially rewarded for improvement [24]. Similarly other authors planned new financing around care groups [35] and operating units [36]. In Canada an ‘Alternative Payment Plan’ enabled physicians to delegate tasks to teams, allowing them to spend additional time with their more complex patients [40]. Incentives are required for innovative and alternate models promoting inter-professional teamwork, e.g. to fund keeping people at home, out of hospital and evaluate better integration of primary/secondary care [26,36,39,40]. The role of meso-level organisations in the devolution of incentives has yet to be determined [26]. Incentives are required to encourage participation in developing integrated care [17], with evidence supporting weak incentives for integrated care hindered change and sustainability [35,36]. Aligning incentives to increase accountability for cost and quality across the continuum requires data for reporting and auditing [35,38,39]. Although pooled budgets and incentive structures, such as equitable funding distribution, for services for a given population across a finite geographical area has been supported in the literature [16,29,30,37,39] and suggest improved partnership working [30] no significant clinical or financial outcome data is available at this time.

### Population focused care

Integrated care requires a change in focus, from health services delivered by separate units to care that can be provided across organisations for a population [16]. Thirteen studies considered options for providing population focused care. Enrolled/registered populations with primary care practices across a region [13,25,26,34], and health plans in the US [24,36], provide a complete and confidential record of present health status of individuals within parts of the health system. However, a geographical population health focus is considered essential to achieve a fully integrated health system, maximising patient accessibility and minimising duplication of services [16,29,30,35,36,38-40]. Both the Integrated Care Pilot in England [30] and the Accountable Care Organisation in the US [38] manage cost and quality for defined populations but data around care outcomes and efficiency of these models is yet to be presented.

### Measurement

Using data as a measurement tool for both quality improvement and redesign was found in twelve studies. Success of integrated health systems is felt to depend on a strategic focus on quality improvement by systematically examining data at different levels and mapping clinical processes to identify gaps, causes of variation and to test improvement [13,18,24,28,35,36,39]. Adopting an improvement methodology that evaluates the impact of new care models and gleans lessons for subsequent innovation allows efforts to both benefit from, and systematically add to, overall ‘innovation architecture’ that ‘makes the next care-model design better, faster, or cheaper’ [36,39]. Using data from interventions this way creates a learning tool to drive change and support quality with an emphasis on health system performance and accountability [17,18,24,32,38]. One US site developed a clinical integration strategy focusing on data driven analysis and prioritisation of value-added processes which has produced a variable cost saving of US$15 million on a US$4 million investment [24]. Also, the accreditation program from Accreditation Canada, Qmentum, has placed a greater emphasis on health system performance and accountability [17]. Finally, a collaborative approach to measuring performance enables clinicians and managers to see issues from a patient perspective with measurement of targets beyond organisational boundaries [13,17,29]. By incorporating a quality assurance program involving a multi-disciplinary physician group, improvement in the quality of patient care was reported by resolving issues from across the continuum and not simply moving them downstream [17].

### Professional development

Professional development to support joint working was supported by half of the included studies in this review. The first of these was training to support new way and align cultures. Training addresses quality improvement theory, measurement and tools, healthcare policy and systems, and leadership provided to all clinical staff and leaders [13,18,24,29,39]. It is estimated an Advanced Training Program in the US has yielded a 4-to-1 return-on-investment (ROI) [24]. Secondly, inter-organisational and inter-sectoral multidisciplinary professional development underpins integrated clinical care. Clearly identifying roles and responsibilities and using/developing guidelines across the continuum creates the skill set to meet community need, context and incentives to promote continuity of care supporting smooth transitions of patients from one type of care to another [25,29,32-34,37,39]. Through inter-professional collaboration and training providers identified areas where they could share resources and work more effectively with others [40] and enable all staff to have expanded clinical roles [33].

### Community/patient engagement

Eight studies discussed the need for community/patient engagement in developing an integrated health system. Integrated health systems should be easy for patients to navigate, and research supports the importance of involving, and being representative of, the communities served as well as encouraging them to participate [13,18,36,39]. In England to make the service patient-centred, the move was from ‘delivery of services’ to ‘partnership for better health’ [25]. Consumers are presented with opportunities for input on various levels [39] such as, individual patient/carer experience and satisfaction to improve performance [25,29], to community forums [40] and large public meetings to present health plans and seek views about what people wanted from the new health care system [35].

### Innovation

Seven studies where change had occurred acknowledged the need to ensure adequate resources are available to support innovation. In some cases there has been a dedicated neutral space where professionals collaborate to deliver innovative services and patients access self-care support and education programmes [29], as well as innovation teams focusing on developing and refining an infrastructure that can adapt to new evidence, efficiently and rapidly translate that evidence into care delivery [36]. In other cases the ability to recognise, develop and use innovative approaches are a result of targeted strategies around care coordination and transitions, chronic care optimisation and illness prevention, transformation of acute episodic care, workforce shortages and role flexibility, and engagement of patients [13,18,29,33,34,36]. Finally the ability to fund flexibly supports innovative care development [18,36,40]. For example, the ability to create policies that encourage greater organisation of care delivery and collaboration among payers and providers has fostered the propagation of innovation that has enhanced value [36].

### Key enablers and barriers

Integration is about relationships between people [28,29] which need to be worked on and built if integration is to be meaningful and sustained. Key factors to achieving this include leadership, a vision that remains centre stage focusing on patient safety and quality care and commitment to partnership [17,18,24-26,28,29,36,39,40]. However, in undertaking integration initiatives a number of significant barriers were identified. Firstly, the most significant is the existence of conflicting aspirations of different parts of the system and the need to balance the interests and values of all stakeholders involved in the continuum of care [17,24,25,28,32,33,39,40]. Additionally, to determine a governance model that serves the interest of the community while preserving the autonomy of individual organisations is a challenge which needs to be addressed [16-18,35]. Secondly, macro-level reforms alone are insufficient to deliver integrated care as they need to be linked to meso-level and micro-level reforms [26,35]. Finally, a feature of much of this work has been the failure to document, evaluate and share lessons learnt in trying to effect change [26,31,34].

### Limitations

Retrieval of qualitative studies form biomedical databases remains a challenge particularly for an area not widely published. We used a wide combination of search terms to optimise the reach and searched grey literature and on-line resources to maximise our reach. The definition of integrated healthcare governance is very broad heading and there is variation internationally. We used a broad search strategy to account for this and the testing of these elements in different settings may yield further validity to both the concept and the definition.

We only reviewed papers published in English and may missed potentially relevant titles and articles published in other languages.

Determining quality for complex literature, particularly qualitative research is challenging as no hierarchy of study design exists [41] and author interpretation may cause bias. Qualitative synthesis is the most difficult to describe and is, potentially, the most controversial, since it is dependent on the judgement and insights of the reviewers. We included one reviewer (SW) who had no prior experience in this area of research to manage potential bias.

## Conclusions

Many countries are looking to integrated care to help deliver more cost effective high quality care. Various examples of successful primary/secondary care integration are reported in the literature and all have focused on a combination of some, if not all, of the ten elements described in this paper and there appears to be agreement that multiple elements are required to ensure successful and sustained integration efforts. This review builds on previous systematic reviews [16,37,39] which individually all identify some of the elements but not all. Also, whilst there is no one model to fit all systems, these elements provide a focus for setting up integration initiatives which need to be flexible to be adapted to local conditions and settings.

There are a few ‘big ticket’ items in taking integrated primary/secondary care governance forward. In relation to joint planning how do we make it meaningful – what structure can manage risk across the continuum, who is accountable i.e. where does the buck stop and with whom? The adoption and use of shared electronic health records will cost before it pays but is pivotal to managing performance and quality across the continuum – how do we link clinical improvement across disparate systems? Within the change process how do we link macro, meso and micro reform? We suggest the ten elements as a starting point along with a realistic synthesis evaluation of the process, as we cannot know what we do not measure. Finally, how do we incentivise integrated care? Pooled funds and sharing in savings seem like a good idea but in, for example, Australia, New Zealand and Canada, complex funding divides between primary and secondary care systems continues to be a significant barrier. Looking forward, what the system needs now is political will, leadership at macro, meso and micro level, and willingness to invest and share risk in determining new models’ fit for the future.

What is critically missing is empirical evidence that integration at scale across primary/secondary care provides the clinical, financial and system benefits it aspires to and how the elements described help achieve this. Another limitation of the literature is the lack of reported perceptions of the impact or experience of integrated service development from patients, health professionals or policy makers. The lack of research in this area is one that needs to be addressed urgently given the drive and expectation of integrated care in the future.

## Competing interests

The authors declare that they have no competing interests.

## Author contributions

Research design was developed by CN. CN reviewed papers for inclusion or exclusion. CN wrote the paper and CJ, JM critically reviewed all drafts and final copy. All authors read and approved the final manuscript.

## Pre-publication history

The pre-publication history for this paper can be accessed here:

http://www.biomedcentral.com/1472-6963/13/528/prepub
