# Erratum to: A governance model for integrated primary/secondary care for the health-reforming first world – results of a systematic review

**DOI:** 10.1186/s12913-017-2444-4

**Published:** 2017-08-17

**Authors:** Caroline Nicholson, Claire Jackson, John Marley

**Affiliations:** 1 Discipline of General Practice, University of Queensland, Brisbane, Australia; 2 Mater/UQ Centre for Primary Health Care Innovation, Mater Health Services, Level 2 JP Kelly Building, Raymond Tce, South Brisbane, Qld 4101 Australia

## Erratum

Following publication of the original article [1], the author reported that in the results and discussion section on page 4 of 12, line 4 – ‘five from Canada’ should be replaced with ‘four from Canada’.
